# Utilizing a Team Kinesiology Model to Support Rehabilitative Care in Patients

**DOI:** 10.3390/ijerph19042079

**Published:** 2022-02-13

**Authors:** Paulette M. Yamada, Joe Priest

**Affiliations:** 1 Department of Kinesiology and Rehabilitative Sciences, University of Hawai‘i, Mānoa, Honolulu, HI 96822, USA; 2 School of Kinesiology, Tarleton State University, Stephenville, TX 76401, USA; priest@tarleton.edu

**Keywords:** kinesiologists, internship, long-term rehabilitation, quality of life

## Abstract

An approach that provides a standardized way of continuing rehabilitative care to help patients return to their lives and activities of daily living (ADL) in an economical and efficient manner is the Team Kinesiology Model (TKM). Many patients who are given a life-altering diagnosis (i.e., paralysis due to spinal cord injury, cerebral palsy, or cancer) are unable to return to employment, their family or a pre-diagnosis quality of life (QOL) given the current health care resources. This is a longstanding, and urgent problem as population aging and rising multi-morbidity is projected to negatively impact all regions of the world. Utilization of mid-level rehabilitation services is a proposed method to increase accessibility to all populations, including those of lower socioeconomic status or minority populations. Capitalizing on this idea, we describe two different programs that use the TKM to provide rehabilitative services to patients who were diagnosed with nervous system dysfunction or cancer. This model benefits the patient by improving physical fitness, psychosocial function, and QOL. Furthermore, we provide specific examples that show how this approach could have further-reaching impacts on society, education and research. Integrating kinesiologists and TKM in health care could assist in workflow, long-term health surveillance, rehabilitation and improvement of QOL.

## 1. Introduction

What happens to an individual after s/he is diagnosed with a life-altering disease such as stroke, cerebral palsy (CP), multiple sclerosis (MS), spinal cord injury (SCI), spina bifida, or cancer? The patient receives acute medical care for treatment of the disease to address survivability and functional outcomes [1]. Those with access to health care receive physical therapy to address side effects associated with the disease, which helps patients to exist within their usual environment [1]. However, in many circumstances, inpatient or outpatient therapy care is not sufficient as this approach requires patients to follow through with therapy work at home. Some fail to complete the assigned therapy homework due to lack of direction or motivation, or patients are unable to do so independently [1,2]. A standardized way of continuing rehabilitative care to help patients return to their lives and activities of daily living (ADL) does not exist. The current health care model (in the United States, USA) works for individuals who rebound quickly, regain functional capacities and return to their jobs, families, and hobbies. However, this model does not work for all.

Consider the rehabilitation journey of a SCI patient after being discharged from an American-based hospital without functional control and being unable to walk or stand independently. S/he will be provided with physical therapy for approximately 3 months, until health coverage runs out [3], but not long enough to regain independence. This results in regression in physical strength, joint flexibility, and overall health due to physical inactivity. The patient will depend on a caregiver (partner, child, or a professional that comes with a price tag) for dressing, bathing, toileting, and food preparation—basically most, if not all self-care tasks. S/he will lose employment due to loss of physical function, and the emotional toll is significant. Learning how to adapt to a life in a wheelchair and not knowing how to return to exercise or sport are barriers to well-being [3].

Patients with nervous system dysfunction have limited voluntary control of their muscles—and, in some cases, their caregivers have been advised to seek hospice care because there is no alternative [2]. Without access to affordable, long-term rehabilitation, especially in the USA, SCI patients are at an increased risk for fatal complications, i.e., pressure sores and urinary tract infection [4]. This may explain why some providers suggest to seek hospice care even when patients are still very much alive in their minds. Patients with severe spinal cord lesions do not have the capacity to connect voluntary movement with the nervous system. Expression through words may be difficult; voluntary muscle control is inhibited [2], yet their minds are active and processing.

Real-life examples have been eloquently described, where in one case, a 49-year-old quadriplegic cowboy (paralysis of all 4 limbs) gained 70 pounds after his injury and, as a result of the skeletal muscle dysfunction associated with SCI, became addicted to methamphetamines [2] (pp. 120–123). After two years of supervised high-repetition training at the Laboratory for Wellness and Motor Behavior (LWMB, Stephenville, TX, USA), even though his C6 injury persisted, he lost 70 pounds and learned how to swim in the university Olympic pool. With rehabilitation and his “never-quit” bull riding mentality, he was able to swim a continuous mile. He won the gold medal in the 200 m backstroke at the Paralympic Games and then subsequently competed in the Tokyo Paralympic Games in table tennis. This athletic achievement in itself is remarkable, but the fact that he stopped taking drugs makes it even more phenomenal. Pain and spasticity have been reported to be significant, physical barriers for recovering SCI patients [5], and he was able to get off of the addictive medication. SCI patients are often prescribed baclofen to control clonus, or involuntary muscle spasms. A side effect of this drug is muscle weakness. With rehabilitation, this patient was able to regain motor control and skeletal muscle function in his previously atrophied muscles. Physiological evidence and rationale which explains physical improvements with repetitive, passive movement of arms and legs have been previously described [6]. As a result, he regained coordination of his remaining normally-innervated muscles, and most importantly, he reintegrated into the community.

Providers share that there is nothing more that they can do because in the US, long-term survivorship care for these recovering populations does not exist [2]. In able-bodied populations, they are given general physical activity guidelines, but this is where long-term rehabilitative care stops, particularly in the USA. In the case of patients diagnosed with cancer, treatments result in severe deconditioning, increased risk of metabolic disease, cardiovascular dysfunction, osteoporosis, and neuropathy [7], which not only reduces health, but reduces the likelihood that patients return to activities which used to bring fulfillment and enjoyment [8].

While countries with universal health care may have access to long-term rehabilitation, availability of a workforce is rare [1]. In many of these countries, demand for rehabilitation services outweighs capacity and there is an insufficient number of programs [9,10,11]. Worldwide, rehabilitative services are limited by the availability of human resources; rehabilitative services are not widespread, and countries with the highest need have the lowest number of professionals [12]. In other words, even in the presence of health care coverage, the service itself may not be available.

A pre-existing condition compounds the effect of physical inactivity by increasing the risk of co-morbid and non-communicable diseases (NCDs), such as coronary heart disease, metabolic disease, colon and breast cancers [13]. This is a worldwide problem [13]. In fact, the prevalence of multi-morbidity of low- and middle-income countries approach those of wealthier countries [14]. There is a need for a better care continuum as this problem will persist with greater urgency in the upcoming years [1]. The prevalence of NCDs is increasing and has already risen by 13.7% worldwide in the past decade [15]. This will have the most impact on patients of low-to-middle incomes because it is the economically disadvantaged populations who have limited access to health care [1]. It is critical to use new approaches for a growing problem.

Quality of life (QOL) declines and in some cases involving paralysis, the ability to resume life seems impossible. Some patients are forced into early retirement. Consider the impact of a dwindling workforce (complemented by increasing health care costs) on the health of the economy. While the Team Kinesiology Model (TKM) has been applied in the USA, this model can be applied within international communities. Worldwide, in 2019, there were 703 million people of retirement age (65 years) and this number is expected to double to 1.5 billion in 2050. By 2050, 1 in 6 people worldwide will be over the age of 65 years [16]. Population aging is a global phenomenon which affects all countries worldwide. From a health care cost standpoint, an aging population with greater medical needs will increase health care demands and costs, especially if access to medical care expands to universal health coverage [1]. The increase in old-age dependency ratio (OADR, the number of people ≥65 years per 100 persons of working age (20–64 years)), is projected to impact all regions of the world. The rising OADR negatively impacts the workforce and economy.

Then, factor in the number of people who have lost the ability to work, but could work with some level of independence, given the proper care. Take for instance, a stroke-survivor, a former city mayor and real estate executive, who could not walk, talk, or swallow [2] (pp. 140–144). After his release from conventional medical care and physical therapy in the USA, he received 1.5 years (3 academic semesters) of supervised training in the LWMB, where he accomplished over 86,000 repetitions of his paralyzed limbs. He returned to work, sold over 12 million dollars of commercial real estate, and was awarded the Top Commercial Real Estate Producer. The following year, again, he was the Top Producer, but this time he walked up to the podium to receive his award.

The impact of rehabilitating the older generation would not be contained just to the economy, but it will also impact how well we preserve our community and culture [17]. Older people have a sense of purpose and engagement [17] and strong desire for social connectedness, and participation and integration in community [18]. Even more, with life-long experiences and awareness, the older generation has the capacity to help the younger generation develop talents and knowledge [19].

The purpose of this paper is to highlight an approach that could enhance the availability of rehabilitation, while also widening the scope of rehabilitative services. Herein, we describe two different programs that use TKM strategies in different populations and then discuss its potential societal, educational and research impacts. We close by describing initial steps that should be considered when building a TKM program.

The TKM is an approach which solves a longstanding and quickly accelerating issue. This approach is not new. It is just not streamlined in a way that it could be widely adopted. Here, we describe how this model is used into two different settings, (1) a university-based lab, converted classroom, named the Laboratory for Wellness and Motor Behavior (LWMB, located on the campus of Tarleton State University, Stephenville, Texas) which serves patients with nervous system dysfunction (i.e., MS, SCI, CP, stroke, spina bifida), and (2) a rehabilitation hospital which serves adult patients diagnosed with cancer through a program called iCare (implementation of a cancer rehabilitation program). These programs were started at different times, work with different populations, but use similar workflows. The first began in 1994 by a then Associate Professor in Kinesiology, and the latter in 2017 which involves collaboration with an Assistant Professor of Kinesiology at the University of Hawai‘i. Both rehabilitation programs recruit senior-level Kinesiology students to volunteer as exercise specialists who work one on one with their assigned patients. These student interns provide exercise rehabilitation free of charge, whilst gaining invaluable clinical experience. Despite operational differences, strikingly, both sites report the same consistent outcomes: (1) improvement in patient health and QOL [6,20,21], (2) enhancement of student education experiences, and strengthened clinical and professional skills [22], (3) promotion of quality research [20,22] (personal observation), and (4) a strengthened sense of community [2] (personal observation).

## 2. Team Kinesiology Model (TKM)—Integrating Internships into Survivorship Care

We propose that kinesiologists be integrated as long-term survivorships care professionals within the current health care model. Kinesiologists are professionals who are trained in assessing human movement, function and performance; and are knowledgeable in the effects of exercise in various applications, i.e., biomechanics, neuroplasticity, motor learning, psychosocial and physiological function. This profession is not licensed and does not have a professional designation (in many countries including the USA) which makes this term ambiguous. For this paper, we refer to kinesiologists as individuals who have received or are on their way to receiving a bachelor’s degree in Kinesiology.

Currently, patients receive medical care, and then are referred to physical or occupational therapy. Since these therapy visits are determined by the presence of an acute diagnosis and the number of treatment appointments are limited, chronic dysfunction such as paralysis, impaired cardiovascular function, or pain is not adequately treated through this workflow [1]. Kinesiologists should be tasked to provide exercise rehabilitation, or exercise that meets recommended guidelines, which targets all components of fitness, resulting in coordinated improvements of all major physiological systems (i.e., cardiovascular, pulmonary, musculoskeletal, metabolic) [23]. This model is similar to tertiary prevention programs, where rehabilitation helps to manage the lasting effects of an illness such as dementia and Parkinson’s disease [24,25,26]. Two differences between tertiary care and TKM are that the current model provides a means to serving populations who have been historically excluded from prevention strategies, and TKM could be implemented in areas with limited resources.

As part of patient care workflow, kinesiologists may refer patients back to their providers if abnormalities arise. For example, in the oncology realm, since cancer treatment negatively affects the cardiovascular system, kinesiologists may detect sudden changes in blood pressure or the onset of atrial fibrillation. In this case, the patient would be referred to their provider. If symptoms of lymphedema are detected, the patient would be referred to physical therapy. These examples, which have occurred in our program, illustrate how TKM can be incorporated into health care and how it facilitates fluidity between health care levels. It has been internationally recognized that *Exercise is Medicine* and it is time to integrate this piece into practice [27,28].

After success in long-term exercise rehabilitation programs, when patients feel comfortable with their newfound knowledge about how to exercise, and they achieve a fitness level which enables them to take charge of their exercise program, they may choose to leave the program. This process ensures that patients are capable of resuming their ADLs, have reached a QOL consistent with pre-medical diagnosis, and may even feel comfortable joining a commercial fitness center or group fitness class. They return to being productive members of the community, and return to their employment and to their families. It has previously been advised that the use of a transdisciplinary approach and the addition of a mid-level rehabilitation workforce may increase accessibility to all populations, including those of lower socioeconomic status or minority populations [1].

**Figure 1:**
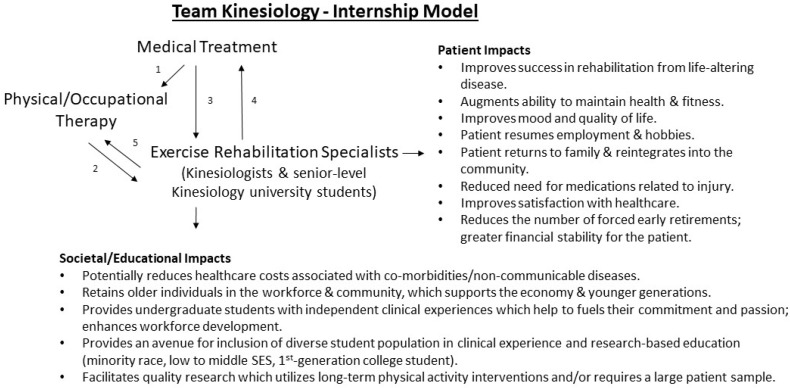
Proposed Team Kinesiology Model to enhance survivorship care. (1) Medical providers refer patients to therapy for the treatment of an acute diagnosis, where they receive a few visits at a time or rehabilitation for a short-term period; (2) Therapists refer patients to kinesiologists trained in working with the specific patient population (i.e., spinal cord injury, stroke, cancer patients) who help patients complete therapy homework and/or provide long-term exercise leadership to mitigate chronic conditions or dysfunction; (3) Medical providers may refer patients directly to kinesiologists for exercise programming and leadership; (4) kinesiologists refer patients back to medical providers when required, i.e., changes in health; (5) kinesiologists refer patients to therapy when required, i.e., emergence of movement-related pain. SES (socioeconomic status).

In the figure below, we provide an overview of the TKM (Figure 1).

Barriers of long-term care include the inability to provide exercise rehabilitation due to insufficient funding [29], limited insurance benefits for rehabilitation therapy [3] and a lack of trained specialists who are knowledgeable in exercise programming for patients [30,31,32]. The LMWB serves patients who drive as far as 50 miles to obtain services, illustrating a need for alternative care options. One way to balance these challenges is to integrate student internships. In both of our programs, we utilize senior-level Kinesiology interns who apply their academic knowledge in a clinical setting to provide free exercise training to patients. In this case, students gain practical clinical experience, which builds the workforce of trained exercise specialists and patients receive care that they would not normally receive. Additionally, students also use this internship as their capstone project, which fulfills their Kinesiology degree program requirements. Specific details of how student training and how the internship is facilitated has been previously described [22].

After comparing notes from our independent rehabilitation clinics, which serves diverse populations and utilizes senior-level undergraduate Kinesiology interns, we discovered that both of our programs have identical, meaningful impacts on society, health, education and research. This discovery is much too significant to keep to ourselves. Below we describe the impact of TKM that extends beyond each patient that we treat and each student we mentor.

## 3. Societal Impacts

### 3.1. Health Care

From this perspective, patients have access to services that they would not normally receive because they are not a part of standardized care (at least in the USA). Even with financial resources, these services are not readily available as it requires specialized exercise equipment and a specialized workforce, issues which are not easily remedied. This model develops a workforce of specialists with the expertise required to work with special populations and provides ongoing exercise rehabilitation.

As we observe from our independent but similarly modeled programs, patients who exercise reduce medications dosages or quit taking them altogether (i.e., baclofen, Lioresal™ (Saol Therapeutics, Roswell, GA, USA), often medically prescribed to alleviate muscle spasms). High-repetition exercise of paralyzed limbs has been reported to reduce these spasms, thus eliminating the need for and side effects of loss of muscle tone, weakness, drowsiness, confusion, as well as interaction with other drugs [33].

In the realm of cancer, clinical treatment leaves patients muscularly deconditioned (due to physical inactivity), increases the risk of cardiovascular dysfunction, metabolic disease and osteopenia [7]. Patients who undergo 12 weeks of exercise training, following the exercise recommendations for this specific population [34], have improved fitness, health and psychosocial outcomes [35,36]. They report that they “feel stronger, more coordinated,” and they “have more energy and sleep better” [20]. Reduced medication use, improved mood, and return to ADLs reduces the burden on the health care system, emotionally as well as financially. During the COVID-19 pandemic, patients reported greater distress, especially those who experienced persisting symptoms related to co-morbidity [37]. Exercise helps to remedy these effects.

Anecdotally, one oncologist who refers patients to our program shared that patients enrolled in our exercise program have more energy, better sleep and often tolerate their breast cancer treatments better because their arthritis or stiffness symptoms are improved with exercise. Treatments that are given for breast cancer can cause many side effects. Exercise provides a non-medication approach for breast cancer care and is routinely recommended for all of her breast cancer patients. Even more, those patients who exercise seem to be overall better adjusted to the pandemic-related stresses than those who do not exercise [38].

The TKM asks patients to visit the clinic or lab multiple times each week to exercise and they received tailored, prescriptive exercise programs. The personal one-on-one interaction with their personal exercise specialist provides many opportunities to detect symptoms that may otherwise go unnoticed. These “catches” may reduce the emotional and financial burden on patients and their health care providers, i.e., changes in blood pressure or heart function. This example shows how this type of program could potentially improve the way patients perceive the efficacy of their health care. This model could add another layer of heath surveillance, reducing emotional and financial distress as patients have the opportunity to learn about health changes in the early stages.

Another standout example is about a girl who was born with spina bifida and was unable to walk [2] (pp. 132–137). Many of her friends who had the disease had no alternatives to improve their health, so they lost hope. They experienced further degeneration associated with lack of physical activity, became depressed, and died. In the LWMB, in three academic semesters, Kristin completed 130,000 arm/leg steps on the NuStep™ (Ann Arbor, MI, USA), and celebrated by walking a mile with crutches. After her incredible rally, she no longer required medical care for spina bifida. Together, these examples demonstrate the large potential impact TKM could have on health care costs, patient satisfaction with health care and workload of medical providers.

### 3.2. Humanity

A sense of community and social connectedness is associated with increased survival [39]. Social connection positively and significantly impacts mood and psychological health [39]. In fact, low social interaction has a similar health effect as “smoking 15 cigarettes/day and being an alcoholic,” and is more harmful than not exercising and is twice as harmful as being obese [40]. When individuals have the opportunity to practice empathy (the functional dimension of social support), in the case of sharing similar experiences, this improves mood [41], depressive symptoms and QOL [42,43]. Programs which foster social interaction is an essential component to patient health and QOL.

Not surprisingly, exercise itself has the same boosting effect on mood [44,45,46,47]. The socialization associated with exercise may have powerful [21] or even synergetic benefits on well-being [20]. Not only does exercise help patients to feel more connected and reintegrate into a society from which they had been isolated (especially in patients with limited mobility), but this feeling of community benefits all who are involved, including program administrators, faculty and interns. Providing long-term rehabilitation is especially essential in the case of SCI patients, as it has been reported to serve as the “only place to turn in times of need” [2,48]. TKM supports an extended network of empathy, connectivity and hope.

This connection impacts the students as well. Through their internship, students realize their capabilities, and they learn how to apply their knowledge and skills [22]. TKM provides an opportunity for students to prove their capabilities to themselves, as they are responsible for the progress of their patient. During the internship, students meet their patient multiple times each week for a minimum of one academic semester, which means they develop profound rapport with their patients. As they provide exercise leadership, they become a supportive counselor, teacher, confidant and friend to their patients. Students adjust to these new roles and as a result, they begin to see themselves in a new light and their confidence increases. This experience creates esteem, a sense of fulfillment and the understanding of how they can positively impact their community [22]. Having this understanding as they close the chapter on their educational career and embark toward their goals underscores their confidence and capability to contribute to their community in impactful and meaningful ways. These experiences fuel students’ passion, bridge their commitment to society, and help them to reflect on the ways they are connected to community. This internship is more than just a capstone project.

Culture and diversity are inherently built into TKM. This internship model is an opportunity equalizer. We represent universities who embrace racial diversity and thus many of our students come from diverse backgrounds and varying socioeconomic status (SES). With regard to the University of Hawai‘i interns, 12% and 82% report that they are of low and middle SES, respectively (unpublished data). Even more, 70% of the interns are 1st-generation college students (unpublished data). Thus, we have the opportunity to provide exceptional educational experiences to all students. The in-depth training will be of great value as students not only gain content knowledge, but they are able to hone their professional skills (related to Kinesiology, interpersonal relationships and communication), and build self-esteem and self-efficacy. Altogether, this program creates an empathetic, diverse, capable, and highly qualified workforce.

Not only does diversity spark creativity, which drives innovation, but diversity among students is an asset. Student diversity enhances their ability to work with patients with similar experiences. For example, we partnered a student with a patient with intellectual disabilities. Unbeknownst to the administrators, the student had a family member with similar characteristics. As a result, she was able to organically form a relationship with her patient as shared experiences strengthen these interactions. In other words, diversity of interns will likely facilitate the connection with a diverse patient population. Familiarity and processing fluency though shared similar experiences increases the desirability of a new environment, and this results in the formation of trustworthy connections [49]. The LWMB is located within driving distance of 12 universities, and so a wide net of interns representing diverse backgrounds can be cast.

This “mutual interpersonal emotional support” helps to create a sense of community, as previously shown in the realm of exercise [50]. These connections develop between intern and patient, and also among patients who exercise at the same time and see each other in passing. These bonds are extremely strong, perhaps because of the familiarity of shared experiences. The patients’ concern for each other is just as large as the interns’ considerations for their patients.

## 4. Research Impacts

This model streamlines quality research by facilitating longitudinal exercise studies, which are often inhibited by high cost and/or the ability to provide long-term exercise interventions. Even more, an ongoing program strengthens patient recruitment through the development of referral workflows, and word-of-mouth recommendations.

Randomized control trials (RCTs) can be used within this model. This can be achieved through research collaboration where a research partner enrolls a subset of patients who are then randomized into a waitlist control group (who receives a delayed intervention) or intervention group. RCTs are essential for showing cause and effect of a particular intervention. However, translation to the community remains a difficult aspect. Questions arise such as, “How can we implement what we learned? Will people follow the exercise recommendations?”. The TKM-internship program enables translational research and pragmatic application of scientific findings. There are many different ways to implement this model in a way which benefits the stakeholders.

By partnering with collaborators with common research goals, each party can focus on one area, resulting in an end product that is greater than each individual could accomplish alone. For instance, a gerontologist who desires to study the effect of exercise on the incidence of dementia could partner with a Kinesiology team who would deliver the exercise intervention. The gerontologist would refer his/her patients, and the research project is facilitated with minimal resources; the Kinesiology student gains training and research experience, and instructional faculty benefits from the enhanced curriculum that delivers hands-on experience and research training to students, while advancing his/her own research.

This is model is extremely well suited for faculty or professionals who wish to enhance the scope of their scholarly work, but may have limited time due to heavy teaching workloads. Thirty-one student-led research projects have been presented at state and national professional conferences, based upon findings from the LWMB (available references on request). Collaborative faculty research which were facilitated by the use of this model have been published in peer-reviewed journals [20,22,51,52]. Even more, a group of researchers who have been using the TKM model since 1996 was recently recognized by the Translational Journal of the American College of Sport Medicine for their Paper of the Year, where they provide a standardized exercise care model for cancer patients [53]. TKM is an effective research model, especially for university faculty who will be able to teach a full university load while supervising the lab.

In addition to the benefit of producing quality research, another benefit of this model is that any population could be recruited to allow for research of a multitude of fields (i.e., osteoporosis, metabolic disease, autoimmune disorders). For example, we first started working with adult cancer patients and are currently expanding rehabilitation to pediatric cancer patients through a virtual physical activity program. TKM can be used as a foundation for scholarly growth.

## 5. Educational Impacts

Students are provided firsthand experiences that improves professional development (communication, interpersonal skills), practical skills (exercise program and assessment), clinical skills (ability to think on their feet, respond to changing circumstances). Implementation of this program supports curricular development, where students are encouraged to apply classroom knowledge to a clinical environment with close mentorship, guidance and support. These experiences strengthen students’ applications to graduate-level programs and employment opportunities.

Furthermore, research experiences enhance student education. Research-based education may augment the students’ skills and use of evidence-based practice [54]; develop analytical skills, strengthen persistence and independence; increase self-confidence, and even clarify the student’s career path [55]. Internship experiences empowers students in terms of development of shared experiential learning on how to problem solve, resolve conflict and advocate for others [56]. Even more, as educators, we understand that classroom learning is finite. Yet, we know that science is constantly evolving and in order to be successful in a health-related career, students need to understand scientific literature, cultural differences among patients and how to maximize their interpersonal skills in a way that makes them an effective leader. Providing hands-on experiences strengthens professional development. It is obvious that this internship model enhances educational experiences.

Still, faculty who mentor students in research may have come to realize the substantial effort involved [57]. A benefit of this ongoing program is that research questions can be developed based upon the current patient population or retrospective data. In this respect, the program facilitates undergraduate and graduate research. This model encompasses teaching and research in one entity, which facilitates student research experiences, essentially helping to accomplish two goals with one activity.

## 6. Logistical Considerations for Implementing TKM

First steps of program implementation include locating a gym or exercise room that could be used, determining the patient population that will be served and finding partners who have similar goals (i.e., faculty, clinical directors, academic advisors or instructors of Kinesiology students). The number of patients enrolled at one time will depend on the number of student interns and the size of the gym. The LWMB started in a “closet” with one student turned patient, a recumbent bicycle and one student intern. Starting small is recommended.

When recruiting interns, we have found success in identifying top performers with goals of working in a clinical setting (physical therapy, medical school, and nursing) as they are highly motivated, dedicated and persistent. Keep in mind that student attitudes are “caught and not taught”, so ensuring students have the disposition required to work with patients is important [58]. If an academic advisor can assist in recruiting students, this will ensure a smooth and steady stream of qualified interns. Alternatively, instructors of core Kinesiology classes or lab courses with a hands-on learning environment where in-depth student performance assessment takes place, may be especially helpful in the recruitment process. A key component to TKM is to have access to motivated Kinesiology students.

Next steps include developing the training program for student interns, which should provide specific education pertaining to the patient population. This information could include a review from their prerequisite courses and may also include detailed information that they have not been exposed to during their Kinesiology curriculum. The benefit of this model is that these students have learned exercise-related content during their curriculum. The training material will reinforce what they learned, and also provide considerations for the specific patient population. It is helpful to upload all training material on the university’s online classroom management system which allows reiterative learning. Additionally, intern performance should be closely monitored as to support them in their first clinical experience. This can be achieved through a review of hardcopy documentation of the exercises used and the patients’ exercise responses, a review of weekly written reports which may be a part of the practicum coursework, and/or unannounced observations. These steps are used to evaluate student performance as well as ensure fidelity to the protocol in the case of ongoing research. As with most internships, general and professional liability insurance for students will be required, along with any business agreements that need to be executed with collaborating partners.

Another point of consideration is the duration of participation and the time from this enrollment and measurable improvements. The LWMB allows patients to remain in the program indefinitely, with one patient being a member of their program for 27 years! The iCare program allows patients to enroll for two academic semesters, which allows ample time for achieving fitness and educating patients about exercise, but also helps to maintain a manageable waitlist while keeping pace with the demand. If demand for a program becomes very large, use of group-based exercise is an option [21]. Still, a group exercise format may be too overwhelming for student interns without clinical experience and/or it may not fit the patient population (i.e., LWMB partners multiple interns with a single patient because getting quadriplegic patients setup on the exercise machine requires more than one person).

The LWMB works with patients with nervous system dysfunction, where measurable improvements can be made on average within one academic semester. Severe cases may take years to respond (i.e., regain voluntary control of skeletal muscles to walk and stand from a paralyzed state), whereas, in the cancer patient population, physical performance can be consistently detected within 12 weeks, and patients report feeling stronger, less fatigued/more energetic, and having improved sleep in as little as 3–4 weeks. Keeping these details in mind is important if students plan to springboard their research project off this model and have a timeline for graduation.

The most difficult part of operations is scheduling. As administrators, we match the schedule availability of students and patients to determine the pairing. Communication among administrators, patients and students is critical in determining changes to schedule due to travel, illness and emergencies. On the other hand, the historical beauty of TKM lab activities is that it is self-perpetuating, i.e., next-semester interns replace graduating interns, new patient referrals are continual, and existing patients return as long as they need help or are motivated to continue. It is a fascinating and rewarding experience for all involved.

## 7. Conclusions

Kinesiologists are needed in health care and a TKM approach would assist in workflow, as it has the potential to assist with health surveillance, long-term rehabilitation and improve the QOL of patients. Throughout this paper, we addressed societal, research and educational impacts, which have resulted in substantial benefit in our own programs as seen at the community and university levels. During the COVID-19 pandemic, we have had the opportunity to reassess how we approach run-of-the-mill daily tasks and how we tackle larger issues, such as ensuring that our health care needs are met. We had the chance to change the way we exist in the world. We may have been hesitant to make modifications, but in the end we may agree that some adaptations were beneficial. Some changes were less helpful. The point is that we will not know what will have a substantial impact until we give it a try. What we know to be true is that we have implemented the TKM and we have seen larger-than-expected outcomes. When examining current practices in education, health care and research—is this model worth replicating? TKM is ready for replication, as we depend and consult with each other.

## Data Availability

Not applicable.
